# Seroprevalence of *Toxocara* spp. in Pregnant Women: A Systematic Review and Meta-Analysis

**DOI:** 10.1155/2024/1943353

**Published:** 2024-04-20

**Authors:** Sara Shayanfar, Saeed Bahadory, Ali Taghipour, Masoud Foroutan, Amir Abdoli

**Affiliations:** ^1^Zoonoses Research Center, Jahrom University of Medical Sciences, Jahrom, Iran; ^2^Student Research Committee, Jahrom University of Medical Sciences, Jahrom, Iran; ^3^Department of Parasitology, Faculty of Medical Sciences, Tarbiat Modares University, Tehran, Iran; ^4^Department of Basic Medical Sciences, Faculty of Medicine, Abadan University of Medical Sciences, Abadan, Iran

## Abstract

**Background:**

Toxocariasis is an important health problem caused by the parasitic species *Toxocara canis* (*T. canis*) and *Toxocara cati* (*T. cati*). Prevalence of toxocariasis in pregnant women as a vulnerable population is doubly important, and the aim of this study is to estimate the overall prevalence of toxocariasis infection in pregnant women according to the available reports.

**Methods:**

The present study followed the Preferred Reporting Items for Systematic reviews and Meta-Analyses (PRISMA) checklists. A systematic search was carried out in international scientific databases (Google Scholar, Web of Science, ScienceDirect, Scopus, and PubMed) between 1990 and 2023. The overall prevalence of parasitic infection was estimated with a random-effects model. All analyses (overall prevalence, heterogeneity, publication bias, and sensitivity analysis) were performed with comprehensive meta-analysis (V2.2, Bio stat) software.

**Results:**

Amid the final eleven included studies, based on the random-effects model, the estimation of the pooled prevalence of *Toxocara* spp. was 20.8% (95% CI, 9.8–38.7%). The association between the risk factors of toxocariasis and the prevalence of the disease was not statistically significant.

**Conclusions:**

In the present study, significant prevalence was reported; however, considering the limited number of studies, it seems that the actual prevalence of the disease is higher. Therefore, it seems necessary to monitor this health problem in pregnant women.

## 1. Introduction

Toxocariasis is a zoonotic parasitic infection caused by the larvae of *Toxocara canis* and *Toxocara cati*, which are commonly found in the intestines of dogs and cats, respectively [1]. This infection can occur when humans accidentally ingest the eggs of these parasites from contaminated soils or foods [2, 3]. *Toxocara* spp. infections are most common in regions with poor sanitation and hygiene practices and where there is a high population of stray or infected animals [4]. According to the published papers, it is estimated that around 19% of people worldwide may have been exposed with *Toxocara* at some point in their lives [5].

These roundworms that can cause several diseases in humans include visceral larva migrans (VLM), ocular larva migrans (OLM), and neural larva migrans (NLM) [6, 7]. VLM is caused by the migration of *Toxocara* larvae through the body's tissues and organs, leading to symptoms such as fever, cough, abdominal pain, and liver enlargement [8]. OLM is caused by the migration of *Toxocara* larvae into the eye, leading to vision loss, inflammation, and retinal damage [9]. However, the exact number of people affected by VLM or OLM is difficult to determine due to underreporting and misdiagnosis [10].

Pregnant women who are infected with toxocariasis are at risk of adverse outcomes for both themselves and their unborn babies such as miscarriage, stillbirth, birth defects, and damage to internal organs, especially eyes and brain [11–13]. Also, toxocariasis rarely causes perimyocarditis with cardiogenic shock [14, 15].

Despite the harmful effects on pregnant women, fetuses, and newborns, there is currently no global understanding of the prevalence and associated risk factors of *Toxocara* among pregnant women. To address this gap, we conducted a systematic review and meta-analysis to assess the global seroprevalence of *Toxocara* and their associated risk factors in pregnant women.

## 2. Methods

According to the Preferred Reporting Items for Systematic reviews and Meta-Analyses (PRISMA) guidelines [16], we conducted a systematic review and meta-analysis of relevant studies to determine the seroprevalence of *Toxocara* spp. in pregnant women. Two investigators (SS and SB) systematically explored five international databases including PubMed, ScienceDirect, Scopus, Web of Science, and Google Scholar, with a time frame between 1 January 1990 and 1 November 2023. The search terms used were “[*Toxocara* OR Toxocariasis OR *Toxocara* spp.] and [Pregnancy OR Pregnant women]”.

### 2.1. Inclusion Criteria

Articles were included in the meta-analysis if they met each of the following criteria: (1) all population-based, descriptive, cross-sectional, and epidemiology studies published in peer-reviewed journals and reporting the prevalence of *Toxocara* in pregnant women, (2) full text or abstract in English, and (3) published online between 1 January 1990 and 1 November 2023.

### 2.2. Study Selection and Data Extraction

All eligible studies were screened by SS and SB. After the initial valuation and warranting the existence of extractable information, data were extracted and double-checked by AT and AA, respectively. Any discrepancies were resolved through discussion with the principal investigator (AT). The study's extracted items included the first author name, publication year, age range or mean range, geographical area (including country and city), sample type, applied diagnostic method, contact with a dog, onychophagia, consumption of raw meat, contact with sand, total sample size, and positive cases of toxocariasis in pregnant women.

### 2.3. Quality Assessment

In order to potentially study the quality assessment, the Joanna Briggs Institute (JBI) checklist was used [17, 18]. JBI contains ten questions with four answering options including yes, no, unknown, and not available (NA). This is a star-base scale, and the maximum score a study can obtain is ten stars (one star for each item). Studies with a total score of ≤4 were acceptable and included our study.

### 2.4. Data Synthesis and Statistical Analysis

All data analysis steps were performed by using comprehensive meta-analysis (V2.2, Bio stat) statistical analysis software. The random-effects model-based overall prevalence was estimated with 95% confidence interval (CI) and presented in forest plot. Furthermore, the prevalence of toxocariasis was evaluated. The one-out-remove method was used in the sensitivity analysis to determine the effect of each research on the final outcomes. Publication bias assessment was conducted using Egger's test, and *p* value < 0.05 was considered as statistically significant. Additionally, the study's heterogeneity was determined and reported using *I*^2^ statistic.

## 3. Results

**Figure 1:**
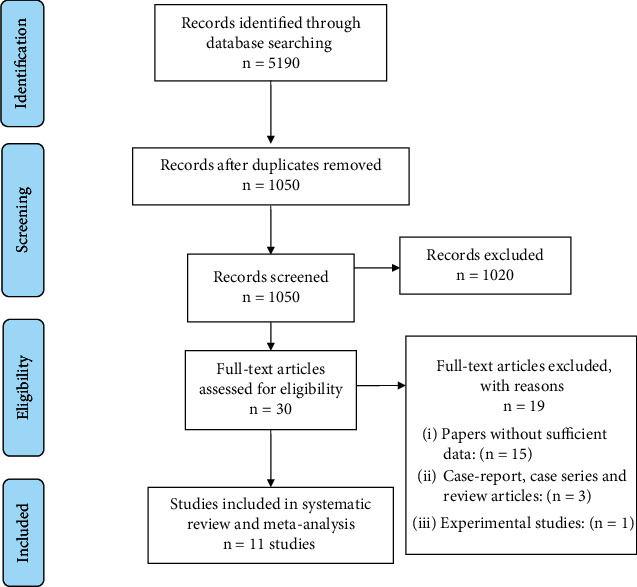
Flow diagram of the study design process.

A flowchart depicting the identification process of qualifying studies is presented in Figure 1. In brief, the systematic search identified 5190 potentially relevant articles. After removing duplicates and/or noneligible papers, 11 articles from six countries across four continents met the inclusion criteria in the systematic review and meta-analysis [19–29]. Serum samples were evaluated by serology methods for *Toxocara*. The main characteristics of each study are shown in Table 1 and Supplementary Table 1.

The results of quality assessment according to JBI for eligible studies are depicted in Table 1 and Supplementary Table 2. The included articles in the present meta-analysis showed an acceptable quality. A total of five studies were available for Asia (1726 individuals), three for South America (871 individuals), two for Africa (441 individuals), and one for Europe (25 individuals). The countries with the highest number of studies were Brazil (three studies) and Iran (three studies). Among the diagnostic methods, only one study used the western blot method and the rest of the studies used the enzyme-linked immunosorbent assay (ELISA) method. A total of 3063 serum samples was evaluated by serology methods for *Toxocara* spp.

**Figure 2:**
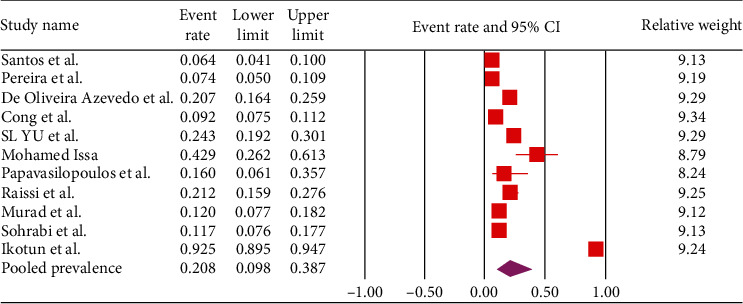
Forest plot of pooled seroprevalence for *Toxocara* spp. in pregnant women.

**Figure 3:**
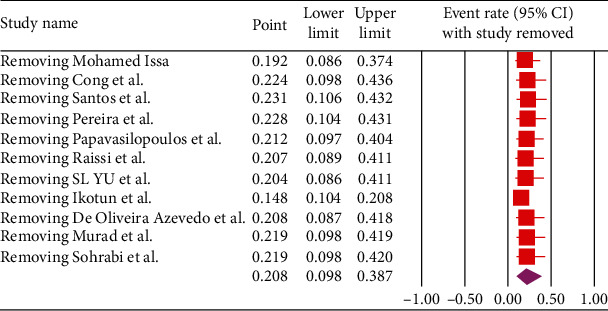
Sensitivity analysis by removing one-by-one studies.

Based on the random-effects model, the estimation of the pooled seroprevalence of *Toxocara* spp. was 20.8% (95% CI, 9.8–38.7%, *I*^2^: 98.28%) (Figure 2). We performed sensitivity analysis by removing one-by-one study method. The results of sensitivity analysis show that the results of meta-analysis are reliable (Figure 3). Although after removing the study by Ikotun et al. [26], the prevalence decreased to 14.8% (95% CI, 10.4–20.8%; Figure 3).

Of the 11 studies, five studies reported contact with a dog, two reported onychophagia, two reported consumption of raw meat, and two reported contact with sand (Supplementary Table 1). No significant association was observed between contact with a dog, onychophagia, consumption of raw meat, and contact with sand and *Toxocara* (OR: 3.2 (95% CI: 0.65-15.67, *I*^2^: 83.83), OR: 1.5 (95% CI: 0.88-2.55, *I*^2^: 0), OR: 1.29 (95% CI: 0.70-2.40, *I*^2^: 5.95), and OR: 1.25 (95% CI: 0.71-2.22, *I*^2^: 0), respectively). Detecting publication bias using the Egger's regression revealed that publication bias was not statistically significant (*p* value = 0.75).

## 4. Discussion

Human toxocariasis is an important one health matter in the developing countries [30]. Although the lack of gold standard and specific clinical symptoms have been the main limitations in estimating the prevalence of toxocariasis close to reality [31], however, the present meta-analysis and systematic review study has addressed the global estimation of toxocariasis in pregnant women according to the available reports. There are several risk factors in the incidence/prevalence of toxocariasis, the most dominant of which are animal contact (mostly dogs and less cats), contaminated soil contact, and so on. The medical importance of this disease is more pronounced in pregnant women because the issue of the mother and/or the fetus infection is raised simultaneously [26]. Analytical findings have indicated a significant prevalence of 20.8% (95% CI, 9.8–38.7%) in the pregnant population; as mentioned, the gold standard method for evaluating toxocariasis has not been defined, so more serology-based (ELISA) and less molecular-based techniques are used for this purpose [26]. In this regard, the remarkable prevalence rate of the disease, in addition to sounding the alarm, can be justified by serological investigations.

Geographically, most of the included studies were from the developing countries of Africa, Asia, and South America. This observation may reflect that these three continents have the majority of low-income and least developed countries (Table 1). Among the countries, three studies were conducted from Brazil and Iran. It is clear that toxocariasis has an undeniable association with the geographical area, so that in many studies, the prevalence in tropical areas has been evaluated more. Interestingly, despite many diseases, the prevalence of toxocariasis is not in direct conflict with the geographical area development level, even it was in line with it according to statistical analysis. One of the risk factors influencing the spread of toxocariasis is contact with animals [32, 33]. In recent decades, with the epidemic of keeping pets and nondomestic animals close to human communities, the transmission of the infectious agent (*T. canis* or *T. cati*) to humans has become possible [32, 33]. However, the correlation results of the risk factor of contact with animals were not statistically significant; unfortunately, statistical analysis and reports of this risk factor have been neglected in studies, but avoiding contact with suspicious animals during pregnancy is an obligatory precaution. As another important risk factor in toxocariasis is contaminated soil contact, the prevalence of toxocariasis in the soil of public places worldwide is estimated at 21% (95% CI, 16-27%) [4], which is a significant amount, and the prevalence in geographical areas, Western Pacific and the African region, has the highest values, and the high prevalence of toxocariasis in pregnant women in these geographical regions is not surprising. Referring to what was mentioned above, the association of this risk factor with the prevalence was not statistically significant.

It is worth noting that the positive in serum titer term of the study subjects does not mean a definite infection and it can even be caused by exposure to an infectious agent; hence, we claim that the estimated values are closer to the apparent prevalence than the true prevalence. Most of the included studies used the ELISA method. Serology is a commonly used method for diagnosing *Toxocara* infection [34]. In this regard, the detection of specific antibodies against *Toxocara* can be done using ELISA or immunoblotting techniques [34, 35].

The strengths of this study include a comprehensive literature search, rigorous methodology, defined clear inclusion and exclusion criteria, quality assessment, subgroup analysis considering risk factors, and sensitivity analysis. However, this systematic review and meta-analysis has certain limitations. First, despite our comprehensive search, there was a paucity or absence of data from different geographical areas, and many of the available studies had limited sample sizes and a lack of data on sociodemographic and/or risk factors. Although we undertook a comprehensive search of the available peer-reviewed literature that had evaluated the seroprevalence of *Toxocara* spp. in pregnant women, we cannot exclude the possibility that some studies may have been missed in the “grey literature.” In addition to the grey literature, the explicit inclusion of the word “pregnant women” in the search term may have reduced the sensitivity of the search. Second, there was high heterogeneity in this meta-analysis. However, we investigated the possible source of heterogeneity by performing sensitivity analysis. Third, the online registration (PROSPERO) failed because the data were already extracted.

## 5. Conclusion

In the present comprehensive study, the prevalence of toxocariasis as a health concern in pregnant women was evaluated as significant value. Considering the small number of analyzable reports and the results of mainly serological evaluations, these estimated values are similar to the tip of the iceberg, and the need for future studies with a larger sample size and the inclusion of risk factors seems necessary. It is suggested that health authorities develop health education programs for women of childbearing age and pregnant women in order to increase their knowledge about *Toxocara* infection. It is also recommended as a preventive health measure against contact with animals and contaminated soil as well as consumption of contaminated and/or suspicious meat during pregnancy.

## Figures and Tables

**Table 1:** Information about *Toxocara* in pregnant women.

First author	Publication year	Age range or mean range	Diagnostic method	Antibodies	Country	Continent	Sample size	Infected by *Toxocara*	QA^∗^
Mohamed Issa	2006	18 to 32	Exoantigen (TEX)-ELISA	IgG	Egypt	Africa	28	12	6
Cong et al.	2014	28.39 (range 18–43)	ELISA	IgG	China	Asia	990	91	9
Santos et al.	2015	13 to 34	ELISA	IgG	Brazil	South America	280	18	10
Pereira et al.	2016	Unknown	ELISA	IgG	Brazil	South America	311	23	9
Papavasilopoulos et al.	2016	Unknown	ELISA	IgG	Greece	Europe	25	4	6
Raissi et al.	2018	30.2	ELISA	IgG	Iran	Asia	189	40	10
SL YU et al.	2020	26.5	ELISA	IgG	China	Asia	235	57	9
Ikotun et al.	2020	31.1 (range 20–43)	Western blot	IgG	Nigeria	Africa	413	382	10
de Oliveira Azevedo et al.	2021	14 to 43	ELISA	IgG	Brazil	South America	280	58	10
Murad et al.	2021	18 to 39	ELISA	IgG	Iran	Asia	150	18	10
Sohrabi et al.	2022	20 to 50	ELISA	IgG	Iran	Asia	162	19	9

^∗^QA: quality assessment.

## Data Availability

The data used to support the findings of this study are included within the article.

## Supplementary Materials

Supplementary 1 Table S1: main characteristics of all eligible studies reporting prevalence of *Toxocara* spp. in pregnant women.

Supplementary 2 Table S2: JBI critical appraisal checklist for analytical cross sectional studies reporting seroprevalence of *Toxocara* spp. in pregnant women.
