# Supplementary material for: Seroprevalence of Toxocara spp. in Pregnant Women: A Systematic Review and Meta-Analysis
Source: Infect Dis Obstet Gynecol. 2024 Apr 20;2024:1943353. doi: 10.1155/2024/1943353 (PMC11055642; doi:10.1155/2024/1943353)
Supplement: Supplementary 1 — Table S1: main characteristics of all eligible studies reporting prevalence of Toxocara spp. in pregnant women. [file 1943353.f1.pdf]

Supplementary Table 1. Main characteristics of all eligible studies reporting prevalence of *Toxocara* spp. in pregnant women.

First author	Publication year	Age range or mean range	Study design
Santos et al.	2015	13 to 34	Cross-sectional
Pereira et al.	2016	Unknown	Cross-sectional
De Oliveira Azevedo et al.	2021	14 to 43	Cross-sectional
Cong et al.	2014	28.39 (range 18–43)	Cross-sectional
SL YU et al.	2020	26.5	Cross-sectional
Mohamed Issa	2006	18 to 32	Cross-sectional
Papavasilopoulos et al.	2016	Unknown	Cross-sectional
Raissi et al.	2018	30.2	Cross-sectional
Murad et al.	2021	18 to 39	Cross-sectional
Sohrabi et al.	2022	20 to 50	Cross-sectional
Ikotun et al.	2020	31.1 (range 20–43)	Cross-sectional

Type of sample	Diagnostic method	Antibodies	Country	Province/city
Serum	ELISA	IgG	Brazil	Rio Grande do S
Serum	ELISA	IgG	Brazil	Brasília
Serum	ELISA	IgG	Brazil	Brazil
Serum	ELISA	IgG	China	Weihai and Qin[illegible]
Serum	ELISA	IgG	China	Yuhang District
Serum	Exo-antigen (TEX)-ELISA	IgG	Egypt	Unknown
Serum	ELISA	IgG	Greece	Athens
Serum	ELISA	IgG	Iran	Ilam
Serum	ELISA	IgG	Iran	Duhok
Serum	ELISA	IgG	Iran	Mashhad
Serum	Western blot	IgG	Nigeria	Osun state

Continent	Sample size	Infected by *Toxocara*	QA	contact with dog (yes) sample
South America	280	18	10	49
South America	311	23	9	
South America	280	58	10	147
Asia	990	91	9	
Asia	235	57	9	
Africa	28	12	6	
Europe	25	4	6	
Asia	189	40	10	65
Asia	150	18	10	
Asia	162	19	9	
Africa	413	382		

Positive	contact with dog (No) sample s	Positive	contact with cat (	positive
3	231	15		
36	75	22	46	9
29	124	1		

contact with cat (	positive	contact with dog and cat (ye	positive	contact with dog and	positive
176	49				
		20	2	130	16
		37	8	44	0

Onychophagia(yes)	positive	Onychophagia(no)	positive
84	7	196	11
134	39	88	19

Consumption of raw meat(yes)	positive	Consumption of raw meat(no)	positive
62	20	160	38
32	3	118	15

Pets ownership(yes)	positive	Pets ownership(no)	posotive
24	3	126	15

Hands washing before meals(y	positive	Hands washing before meals(n	positive
115	14	35	4

Tap water drinking(yes)	positive	Tap water drinking(no)	positive
122	15	28	3

Contact with sand(yes)	positive	Contact with sand(no)	positive
87	5	193	13
53	17	169	41
