# Supplementary material for: Seroprevalence of Toxocara spp. in Pregnant Women: A Systematic Review and Meta-Analysis
Source: Infect Dis Obstet Gynecol. 2024 Apr 20;2024:1943353. doi: 10.1155/2024/1943353 (PMC11055642; doi:10.1155/2024/1943353)
Supplement: Supplementary 2 — Table S2: JBI critical appraisal checklist for analytical cross sectional studies reporting seroprevalence of Toxocara spp. in pregnant women. [file 1943353.f2.docx]

|  | Supplementary Table 2. JBI critical appraisal checklist for analytical cross sectional studies reporting seroprevalence of *Toxocara* spp. in pregnant women. | | | | | | | | | | | |
| --- | --- | --- | --- | --- | --- | --- | --- | --- | --- | --- | --- | --- |
| First author, reference | | **There is congruity between the stated philosophical perspective and the research methodology** | **There is congruity between the research methodology and the research question or objectives.** | **There is congruity between the research methodology and the methods used to collect data.** | **There is congruity between the research methodology and the representation and analysis of data.** | **There is congruence between the research methodology and the interpretation of results.** | **There is a declaration of the researcher’s cultural or theoretical orientation.** | **The influence of the researcher on the research, and vice versa, is addressed.** | **There is representation of participants and their voices.** | **There is ethical approval by an appropriate body** | **There is a relationship between the conclusions of the study and the analysis or interpretation of the data.** | **QA (total score)** |
| Mohamed Issa | | Yes | Yes | Yes | Yes | Yes | No | No | No | No | Yes | 6 |
| Cong et al. | | Yes | Yes | Yes | Yes | Yes | Yes | Yes | Yes | No | Yes | 9 |
| Santos et al. | | Yes | Yes | Yes | Yes | Yes | Yes | Yes | Yes | Yes | Yes | 10 |
| Pereira et al. | | Yes | Yes | Yes | Yes | Yes | Yes | Yes | Yes | No | Yes | 9 |
| Papavasilopoulos et al. | | Yes | Yes | Yes | Yes | Yes | No | No | No | No | Yes | 6 |
| Raissi et al. | | Yes | Yes | Yes | Yes | Yes | Yes | Yes | Yes | Yes | Yes | 10 |
| SL YU et al. | | Yes | Yes | Yes | Yes | Yes | Yes | Yes | Yes | No | Yes | 9 |
| Ikotun et al. | | Yes | Yes | Yes | Yes | Yes | Yes | Yes | Yes | Yes | Yes | 10 |
| De Oliveira Azevedo et al. | | Yes | Yes | Yes | Yes | Yes | Yes | Yes | Yes | Yes | Yes | 10 |
| Murad et al. | | Yes | Yes | Yes | Yes | Yes | Yes | Yes | Yes | Yes | Yes | 10 |
| Sohrabi et al. | | Yes | Yes | Yes | Yes | Yes | Yes | Yes | Yes | No | Yes | 9 |
